# Supplementary material for: A conserved Mcm4 motif is required for Mcm2-7 double-hexamer formation and origin DNA unwinding
Source: eLife. 2019 Aug 6;8:e45538. doi: 10.7554/eLife.45538 (PMC6701924; doi:10.7554/eLife.45538)
Supplement: Supplementary file 1. [file elife-45538-supp1.docx]

**Supplementary File 1**

**Supplementary Table 1.** Fit parameters for *E*_FRET_ distributions during double-hexamer formation^†^

| Protein | Parameter* | *N*_t_^‡^ | Value  90% C.I.^⌂^ |
| --- | --- | --- | --- |
| Wild type Mcm2-7  (Fig. 5D)  *N*_molec_ = 89 | μ_1_ | 5,300 | 0.029  [0.024, 0.034] |
|  | μ_2_ |  | 0.606  [0.603, 0.609] |
|  | σ |  | 0.123  [0.121, 0.125] |
|  | *A*_1_ (0 – 10 s) | 254 | 0.77  [0.73, 0.82] |
|  | *A*_1_ (10 – 30 s) | 704 | 0.42  [0.38, 0.45] |
|  | *A*_1_ (30 – 200 s) | 4,342 | 0.30  [0.29, 0.31] |
| Mcm2-7^4-178A^  (Fig. 5E)  *N*_molec_ = 114 | μ_1_ | 4,908 | 0.038  [0.034, 0.042] |
|  | μ_2_ |  | 0.468  [0.452, 0.482] |
|  | σ |  | 0.143  [0.140, 0.146] |
|  | *A*_1_ (0 – 10 s) | 314 | 0.80  [0.76, 0.85] |
|  | *A*_1_ (10 – 30 s) | 866 | 0.54  [0.51, 0.58] |
|  | *A*_1_ (30 – 200 s) | 3,728 | 0.96  [0.96, 0.97] |

^†^*N*_molec_: Number of complexes analyzed. Measurements with outlier *E*_FRET_ > 1 or *E*_FRET_ < -0.35 (1% for Mcm2-7 and 4.5% for Mcm2-7^4-178A^ were excluded from fits. ******E*_FRET_ data measured between 0 and 200 s after binding of the second Mcm2-7 were fit to a two-Gaussian mixture model that assumed time-invariant values for the standard deviation (σ) and positions of the low- and high-*E*_FRET_ peaks (μ_1,_ μ_2_).and fractional amplitudes of the two peaks (*A*_1_ and 1 - *A*_1_) that can differ between the three time intervals. ^‡^Number of data points contributing to determination of each parameter value. ^⌂^90% confidence intervals were determined by bootstrapping (250 samples).

**Supplementary Table 2. Genotypes of yeast and bacterial expression strains used in this study**

| **Name** | **Genotype** | **Source** |
| --- | --- | --- |
| ASY1055.1 | MATa ade2-1 ura3-11 his3-11,15 leu2-3,12 can-100  trp1-1 mcm2::hisG [pMCM5-MCM2::URA3] | Schwacha and Bell, 2001 |
| ASY1059.1 | MATa ade2-1 ura3-11 his3-11,15 leu2-3,12 can-100  trp1-1 mcm4::hisG [pMCM5-MCM4::URA3] | Schwacha and Bell, 2001 |
| ASY2157 | MATa ade2-1 ura3-11 his3-11,15 leu2-3,12 can-100  trp1-1 mcm6::HISMX6 [pMCM5-MCM6::URA3] | Schwacha and Bell, 2001 |
| MLy049 | MATa pep4::unmarkedbar1::hisG LEU2::pGAL1/10-MCM10-3xFlag | Lõoke et al., 2017 |
| p11d-tscRPA-30MxeHis6 | Expression construct for yRPA1, yRPA2, yRPA3 | Lõoke et al., 2017 |
| pBL481 | Expression construct for yRFC1, yRFC2, yRFC3, yRFC4, yRFC5 | Gomes et al., 2000 |
| pMM054 | pET15b-6xHis-POL30 | Lõoke et al., 2017 |
| pSKM033 | pGEX-GST-3C-FLAG-CDC6 | Kang et al., 2014 |
| yAE34 | MATa ade2-1 ura3-1 his3-11,15 trp1-1 leu2-3,112 can1-100 bar1::Hyg pep4::KanMX ura3::URA3 pRS306-POL31+POL3 his3::HIS3 pRS303-Pol32-CBP+Gal4 | Yeeles et al., 2017 |
| yAE48 | MATa ade2-1 ura3-1 his3-11,15 trp1-1 leu2-3,112 can1-100 bar1::Hyg pep4::KanMX ura3::URA3pRS306-CBP-Csm3 + Tof1 | Yeeles et al., 2017 |
| yAE71 | MATa ade2-1 ura3-1 his3-11,15 trp1-1 leu2-3,112 can1-100 bar1::hphNT pep4::kanMX his3::pRS303-MRC1-5FLAG | Yeeles et al., 2017 |
| yAS26 | MATa pep4::unmarked bar1::hisG LEU2::pGAL1/10-POL3-FLAG URA3::pGAL1/10-GAL4 | Lõoke et al., 2017 |
| yAS3 | MATa pep4::unmarked bar1::hisG TRP1::pGAL1/10-PRI1+PRI2 HIS3::pGAL1/10-POL1+POL12-3C-FLAG | Lõoke et al., 2017 |
| yKC03 | ade2-1 trp1-1 leu2-3,112 his3-11,15 ura3-1 can1-100 bar1::HisG lys2::HisG pep4::KanMX Mcm2-13xmyc(NatMX4)  his3::pKC16 (GAL1,10-mcm2-∆2-177, Flag-MCM3) ura::pALS1 (GAL1,10-Cdt1, GAL4)  lys::pSKM002 (GAL1,10-MCM4, MCM5)  trp::pSKM003 (GAL1,10-MCM6, MCM7) | This study |
| yKC05 | ade2-1 trp1-1 leu2-3,112 his3-11,15 ura3-1 can1-100 bar1::HisG lys2::HisG pep4∆::KanMX6 Mcm7-V5 (NatMX4) Mcm4-V5 (HphMX4)  his3::pSKM004 (GAL1,10-MCM2, Flag-MCM3) ura::pALS1 (GAL1,10-Cdt1, GAL4)  lys::pKC17 (GAL1,10-mcm4-∆2-181, MCM5) trp::pSKM003 (GAL1,10-MCM6, MCM7) | This study |
| yKC06 | ade2-1 trp1-1 leu2-3,112 his3-11,15 ura3-1 can1-100 bar1::HisG lys2::HisG pep4∆::unmarked­  his3::pSKM004 (GAL1,10-MCM2, Flag-MCM3) ura::pALS1 (GAL1,10-Cdt1, GAL4)  lys::pKC18 (GAL1,10-mcm4-∆2-174, MCM5) trp::pSKM003 (GAL1,10-MCM6, MCM7) | This study |
| yKC08 | ade2-1 trp1-1 leu2-3,112 his3-11,15 ura3-1 can1-100 bar1::HisG lys2::HisG pep4::KanMX Mcm6-13xmyc(NatMX4)  his3::pSKM004 (GAL1,10-MCM2, Flag-MCM3) ura::pALS1 (GAL1,10-Cdt1, GAL4)  lys::pSKM002 (GAL1,10-MCM4, MCM5)  trp::pKC19 (GAL1,10-mcm6-∆2-105, MCM7) | This study |
| yKC09 | ade2-1 trp1-1 leu2-3,112 his3-11,15 ura3-1 can1-100 bar1::HisG lys2::HisG pep4∆::KanMX6 Mcm7-V5 (NatMX4) Mcm4-V5 (HphMX4)  his3::pSKM004 (GAL1,10-MCM2, Flag-MCM3)  ura::pALS1 (GAL1,10-Cdt1, GAL4)  lys::pKC20 (GAL1,10-mcm4-7A, MCM5) trp::pSKM003 (GAL1,10-MCM6, MCM7) | This study |
| yKC10 | ade2-1 trp1-1 leu2-3,112 his3-11,15 ura3-1 can1-100 bar1::HisG lys2::HisG pep4∆::KanMX6 Mcm7-V5 (NatMX4) Mcm4-V5 (HphMX4)  his3::pSKM004 (GAL1,10-MCM2, Flag-MCM3)  ura::pALS1 (GAL1,10-Cdt1, GAL4)  lys::pKC21 (GAL1,10-mcm4-∆175-181, MCM5) trp::pSKM003 (GAL1,10-MCM6, MCM7) | This study |
| yKC11 | ade2-1 trp1-1 leu2-3,112 his3-11,15 ura3-1 can1-100 bar1::HisG lys2::HisG pep4∆::KanMX6 Mcm7-V5 (NatMX4) Mcm4-V5 (HphMX4)  his3::pSKM004 (GAL1,10-MCM2, Flag-MCM3)  ura::pALS1 (GAL1,10-Cdt1, GAL4)  lys::pKC22 (GAL1,10-mcm4-175A, MCM5) trp::pSKM003 (GAL1,10-MCM6, MCM7) | This study |
| yKC12 | ade2-1 trp1-1 leu2-3,112 his3-11,15 ura3-1 can1-100 bar1::HisG lys2::HisG pep4∆::KanMX6 Mcm7-V5 (NatMX4) Mcm4-V5 (HphMX4)  his3::pSKM004 (GAL1,10-MCM2, Flag-MCM3)  ura::pALS1 (GAL1,10-Cdt1, GAL4)  lys::pKC23 (GAL1,10-mcm4-178A, MCM5) trp::pSKM003 (GAL1,10-MCM6, MCM7) | This study |
| yKC13 | ade2-1 trp1-1 leu2-3,112 his3-11,15 ura3-1 can1-100 bar1::HisG lys2::HisG pep4∆::KanMX6 Mcm7-V5 (NatMX4) Mcm4-V5 (HphMX4)  his3::pSKM004 (GAL1,10-MCM2, Flag-MCM3)  ura::pALS1 (GAL1,10-Cdt1, GAL4)  lys::pKC24 (GAL1,10-mcm4-181A, MCM5) trp::pSKM003 (GAL1,10-MCM6, MCM7) | This study |
| yKC14 | ade2-1 trp1-1 leu2-3,112 his3-11,15 ura3-1 can1-100 bar1::HisG lys2::HisG pep4∆::KanMX6 Mcm7-V5 (NatMX4) Mcm4-V5 (HphMX4)  his3::pSKM004 (GAL1,10-MCM2, Flag-MCM3)  ura::pALS1 (GAL1,10-Cdt1, GAL4)  lys::pKC25 (GAL1,10-mcm4-182A, MCM5) trp::pSKM003 (GAL1,10-MCM6, MCM7) | This study |
| yKC15 | ade2-1 trp1-1 leu2-3,112 his3-11,15 ura3-1 can1-100 bar1::HisG lys2::HisG pep4∆::KanMX6 Mcm7-V5 (NatMX4) Mcm4-V5 (HphMX4)  his3::pSKM004 (GAL1,10-MCM2, Flag-MCM3)  ura::pALS1 (GAL1,10-Cdt1, GAL4)  lys::pKC26 (GAL1,10-mcm4-185A, MCM5) trp::pSKM003 (GAL1,10-MCM6, MCM7) | This study |
| yKC16 | ade2-1 trp1-1 leu2-3,112 his3-11,15 ura3-1 can1-100 bar1::HisG lys2::HisG pep4∆::KanMX6 Mcm7-V5 (NatMX4) Mcm4-V5 (HphMX4) his3::pSKM004 (GAL1,10-MCM2, Flag-MCM3) ura::pALS1 (GAL1,10-Cdt1, GAL4) lys::pKC27 (GAL1,10-mcm4-188A, MCM5) trp::pSKM003 (GAL1,10-MCM6, MCM7) | This study |
| yKC20 | ade2-1 trp1-1 leu2-3,112 his3-11,15 ura3-1 can1-100 bar1::HisG lys2::HisG pep4∆::KanMX6 Mcm7-V5 (NatMX4) Mcm4-V5 (HphMX4) his3::pSKM004 (GAL1,10-MCM2, Flag-MCM3) ura::pALS1 (GAL1,10-Cdt1, GAL4) lys::pKC27 (GAL1,10-mcm4-178A, MCM5) trp::pST030 (GAL1,10-MCM6, UbSORT-MCM7) | This study |
| yMH28 | MATa pep4::unmarked bar1::hisG TRP1::pGAL1/10-POL2-3C-5xFLAG URA3::pGAL1/10-DPB3+DPB4-3C-His + pGAL1/10-DPB2-3C-FLAG | Lõoke et al., 2017 |
| yMM016 | MATa pep4::unmarked bar1::hisG LEU2::pGAL1/10-CDC45 | Lõoke et al., 2017 |
| yMM050 | MATa pep4::unmarked bar1::hisG LEU2::pGAL1/10-TOP2-CBP | Lõoke et al., 2017 |
| yMM051 | MATa pep4::unmarked bar1::hisG LEU2::pGAL1/10-CBP-CTF4 | Lõoke et al., 2017 |
| yRH146 | MATa pep4::unmarked bar1::hisG LEU2::pGAL1/10-DBF4-FLAG+CDC7 | Lõoke et al., 2017 |
| yRH154 | MATa pep4::unmarked bar1::hisG LEU2::pGAL1/10-Dpb11-FLAG | Heller et al., 2011 |
| ySK100 | MATa pep4::kanMX bar1::hisG orc1::hisG LEU2::ORC1 LYS2::pGAL1/10-ORC2+ORC5 TRP1::pGAL1/10-ORC3+ORC4 HIS3::pGAL1/10-FLAG-ORC1+ORC6 | Ticau et al., 2015 |
| ySK119 | MATa pep4::unmarked bar1::hisG URA3::pGAL1/10-Δ1-95-CLB5-FLAG+CDC28-His | Lõoke et al., 2017 |
| ySK123 | MATa pep4::unmarked bar1::hisG LEU2::pGAL1/10-Δ1-104-SLD3-3xFLAG HIS3::pGAL1/10-SLD7-VSV-G | Lõoke et al., 2017 |
| ySK127 | MATa pep4::unmarked bar1::hisG LEU2::pGAL1/10-3xFLAG-3C-SLD2 | Lõoke et al., 2017 |
| ySK136 | MATa pep4::unmarked bar1::hisG URA3::pGAL1/10-SLD5 LEU2::pGAL1/10-PSF2-3C-His-FLAG LYS2::pGAL1/10-PSF1+PSF3 | Lõoke et al., 2017 |
| yST144 | ade2-1 trp1-1 leu2-3,112 his3-11,15 ura3-1 can1-100 bar1::HisG lys2::HisG pep4∆::unmarked­ his3::pSKM004 (GAL1,10-MCM2, Flag-MCM3) ura::pALS1 (GAL1,10-Cdt1, GAL4) lys::pSKM002 (GAL1,10-MCM4, MCM5) trp::pSKM003 (GAL1,10-MCM6, MCM7) | Ticau et al., 2015 |
| yST144 | MATa pep4::unmarked bar1::hisG TRP1::pGAL1/10-MCM6+MCM7 HIS3::pGAL1/10-FLAG-MCM3+MCM2 LYS2::pGAL1/10-MCM4+MCM5 URA3::pGAL1/10-CDT1+GAL4 | Ticau et al., 2015 |
| yST161 | ade2-1 trp1-1 leu2-3,112 his3-11,15 ura3-1 can1-100 bar1::HisG lys2::HisG pep4∆::unmarked his3::pSKM004 (GAL1,10-MCM2, Flag-MCM3) ura::pALS1 (GAL1,10-Cdt1, GAL4) lys::pSKM002 (GAL1,10-MCM4, MCM5) trp::pST030 (GAL1,10-MCM6, UbSORT-MCM7) | Ticau et al., 2015 |

**Supplementary Table 3. Plasmids used in this study**

| **Name** | **Description** | **Source** |
| --- | --- | --- |
| pALS1 | pRS305 (GAL1,10-Cdt1, GAL4) | Ticau et al., 2015 |
| pKC01 | pJR164 (TRP1-MCM5promoter-MCM2) | This study |
| pKC02 | pJR164 (TRP1-MCM5promoter-mcm2-∆2-177) | This study |
| pKC03 | pJR164 (NatMX-MCM5promoter-MCM4) | This study |
| pKC04 | pJR164 (NatMX-MCM5promoter-mcm4-∆2-181) | This study |
| pKC04 | pJR164 (NatMX-MCM5promoter-mcm4-∆2-174) | This study |
| pKC06 | pJR165 (LEU2-MCM5promoter-MCM6) | This study |
| pKC07 | pJR165 (LEU2-MCM5promoter-mcm6-∆2-105) | This study |
| pKC08 | pJR164 (NatMX-MCM5promoter-mcm4-7A) | This study |
| pKC09 | pJR164 (NatMX-MCM5promoter-mcm4-∆175-181) | This study |
| pKC10 | pJR164 (NatMX-MCM5promoter-mcm4-175A) | This study |
| pKC11 | pJR164 (NatMX-MCM5promoter-mcm4-178A) | This study |
| pKC12 | pJR164 (NatMX-MCM5promoter-mcm4-181FA) | This study |
| pKC13 | pJR164 (NatMX-MCM5promoter-mcm4-182A) | This study |
| pKC14 | pJR164 (NatMX-MCM5promoter-mcm4-185A) | This study |
| pKC15 | pJR164 (NatMX-MCM5promoter-mcm4-188A) | This study |
| pKC16 | pRS403 (GAL1,10-mcm2-∆2-177, Flag-MCM3) | This study |
| pKC17 | pRS307 (GAL1,10-mcm4-∆2-181, MCM5) | This study |
| pKC18 | pRS307 (GAL1,10-mcm4-∆2-174, MCM5) | This study |
| pKC19 | pRS404 (GAL1,10-mcm6-∆2-105, MCM7) | This study |
| pKC20 | pRS307 (GAL1,10-mcm4-7A, MCM5) | This study |
| pKC21 | pRS307 (GAL1,10-mcm4-∆175-181, MCM5) | This study |
| pKC22 | pRS307 (GAL1,10-mcm4-175A, MCM5) | This study |
| pKC23 | pRS307 (GAL1,10-mcm4-178A, MCM5) | This study |
| pKC24 | pRS307 (GAL1,10-mcm4-181FA, MCM5) | This study |
| pKC25 | pRS307 (GAL1,10-mcm4-182A, MCM5) | This study |
| pKC26 | pRS307 (GAL1,10-mcm4-185A, MCM5) | This study |
| pKC27 | pRS307 (GAL1,10-mcm4-188A, MCM5) | This study |
| pMM068 | pUC19-*ARS1*-11.9 | This study |
| pSKM002 | pRS307 (GAL1,10-MCM4, MCM5) | Kang et al., 2014 |
| pSKM003 | pRS404 (GAL1,10-MCM6, MCM7) | Kang et al., 2014 |
| pSKM004 | pRS403 (GAL1,10-MCM2, Flag-MCM3) | Kang et al., 2014 |
| pST030 | pRS404 (GAL1,10-MCM6, UbSORT-MCM7) | Ticau et al., 2015 |
| pUC19-*ARS1* | pUC19-*ARS1*-3.8 | Heller et al., 2011 |
